# Partner notification and partner treatment for chlamydia: attitude and practice of general practitioners in the Netherlands; a landscape analysis

**DOI:** 10.1186/s12875-017-0676-3

**Published:** 2017-12-20

**Authors:** Ingrid V. F. van den Broek, Gé A. Donker, Karin Hek, Jan E. A. M. van Bergen, Birgit H. B. van Benthem, Hannelore M. Götz

**Affiliations:** 1 Epidemiology and Surveillance Unit, Centre for Infectious Diseases Control, RIVM, Bilthoven, The Netherlands; 2 NIVEL Primary Care Database, Sentinel Practices, Utrecht, The Netherlands; 3 NIVEL Primary Care database, Utrecht, The Netherlands; 4 Epidemiology and Surveillance Unit, Centre for Infectious Diseases Control, RIVM, Bilthoven, The Netherlands; 5 Department of General Practice, Academic Medical Center, Amsterdam; STI AIDS Netherlands, Amsterdam, The Netherlands; 6 Department of Infectious Disease Control, Municipal Public Health Service Rotterdam-Rijnmond, Rotterdam, The Netherlands; 7 Department of Public Health, Erasmus MC—University Medical Center Rotterdam, Rotterdam, The Netherlands

**Keywords:** Partner notification, Partner treatment, Expedited partner therapy, Patient-initiated partner treatment, General practice, Sexually transmitted infections, Chlamydia trachomatis

## Abstract

**Background:**

Chlamydia prevalence remains high despite scaling-up control efforts. Transmission is not effectively interrupted without partner notification (PN) and (timely) partner treatment (PT). In the Netherlands, the follow-up of partners is not standardized and may depend on GPs’ time and priorities. We investigated current practice and attitude of GPs towards PN and PT to determine the potential for Patient-Initiated Partner Treatment, which is legally not supported yet.

**Methods:**

Multiple data-sources were combined for a landscape analysis. Quantitative data on (potential) PT were obtained from prescriptions in the national pharmacy register (2004–2014) and electronic patient data from NIVEL-Primary Care Database (PCD) and from STI consultations in a subgroup of sentinel practices therein. Furthermore, we collected information on current practice via two short questionnaires at a national GP conference and obtained insight into GPs’ attitudes towards PN/PT in a vignette study among GPs partaking in NIVEL-PCD.

**Results:**

Prescription data showed Azithromycin double dosages in 1–2% of cases in the pharmacy register (37.000 per year); probable chlamydia-specific repeated prescriptions or double dosages of other antibiotics in NIVEL-PCD (115/1078) could not be interpreted as PT for chlamydia with certainty. STI consultation data revealed direct PT in 6/100 cases, via partner prescription or double doses. In the questionnaires the large majority of GPs (>95% of 1411) reported to discuss PN of current and ex-partner(s) with chlamydia patients. Direct PT was indicated as most common method by 4% of 271 GPs overall and by 12% for partners registered in the same practice. Usually, GPs leave further steps to the patients (83%), advising patients to tell partners to get tested (56%) or treated (28%). In the vignette study, 16–20% of 268 GPs indicated willingness to provide direct PT, depending on patient/partner profile, more (24–45%) if patients would have the chance to notify their partner first.

**Conclusion:**

GPs in the Netherlands already treat some partners of chlamydia cases directly, especially partners registered in the same practice. Follow-up of partner notification and treatment in general practice needs more attention. GPs may be open to implement PIPT more often, provided there are clear guidelines to arrange this legally and practically.

## Background

Chlamydia trachomatis (hereafter “chlamydia”) incidence remains high in the Netherlands despite targeted control efforts. The estimated report rate was 310/100,000 in 2015 and 272/100.000 in 2010. [1] STI centres provide low-threshold additional care prioritising high-risk groups. Young people under 25 years of age and other high-risk groups can be tested for chlamydia without cost at STI clinics. The general practitioner (GP) still performs the largest number of STI consultations (estimated at two thirds or more) [1]; a minority uses private (self-)care and some get tested in the hospital (gynaecologist) after referral. The number of chlamydia tests has increased over the years but diagnostic rates have not gone down: an estimated 54,000 infections were detected at GPs and STI clinics in 2015: 35,000 at the GP and 19,000 in STI clinics. [1] New routes of chlamydia control need to be explored and integrated in the STI care system.

Effective partner notification (PN) and partner treatment (PT) are essential to prevent re-infection and further spread. Re-infections for chlamydia are common, occurring in 14–20% of cases [2–5].The importance of PN for chlamydia has been demonstrated in several studies [6] and is addressed in all Dutch STI care manuals and guidelines [7, 8]. Persons notified by a sexual partner are at high risk of infection; in Dutch STI clinics the infection rate was 36% among women and 32% among heterosexual men who visited due to notification for chlamydia. [1]

Dutch guidelines advise PN for chlamydia patients (a) in case of symptomatic infections for partners in the period 6 weeks prior to the onset of symptoms and (b) in case of asymptomatic infections for all partners in the last 6 months. According to GP guidelines, [7, 8] PN should be discussed in order to motivate chlamydia-patients to inform their sex partners, or alternatively, GPs should offer to help with notification or refer to other services. The advice is to tell the partner to see a health professional to get tested or to get treated directly. GPs can offer presumptive treatment for a partner presenting for consultation. Online tools for patients and GPs are available: ‘www. Thuisarts.nl’ gives instructions on how to perform PN and ‘partnerwaarschuwing.nl’ [9] can be used to arrange individual PN after diagnosis, but uptake at GPs is still limited [10].

A way to improve partner management is to provide practical tools to simplify procedures for both patients and GPs. A systematic review showed that providing direct treatment for the current sexual partner, so-called Expedited Partner Therapy (EPT), can be an effective strategy. [6] Patients give a prescription or medication to their sexual partners to treat the infection or, alternatively, partners obtain medication from the pharmacy directly. [11] EPT is efficacious in reducing repeated infections in index patients and increasing the number of partners treated. [12–15] The implementation of EPT reduced the chlamydia test positivity in a large scale pilot in Washington State, USA, although this may not have been an exclusive effect of the intervention. [16] The method is used widely in the USA: EPT is permissible in 38 states and potentially allowable in 8 states. [17] Also in the UK, EPT or similarly, Accelerated partner therapy (APT), is seen as a viable option. [18–20]

In the Netherlands, direct PT via the partner, referred to as Patient Initiated Partner Therapy (PIPT), is not officially implemented, because a physician is legally required to have contact with a patient before prescribing medication, to explain the reason for medication and to check medical history and allergies. In 2016, the Project Investigating options for Patient Initiated Contact treatment for Chlamydia in the Netherlands (PICC-UP) started, with the objective to investigate the potential of PIPT as an effective method for improving PT. The project covers other issues of PIPT, including the legal steps needed for implementation. Here we describe an initial landscape analysis on the current level of practice of PN/PT and (potential) direct PT for chlamydia in the general practice, as well as the attitude and opinions of Dutch GPs towards implementation of direct PT, provided it would be possible in future. [21]

## Methods

This study was set up to combine multiple sources to explore PN/PT practices among GPs in the Netherlands, both prescription data (paragraph 1) and questionnaire data (paragraph 2) were used.

### Quantitative data on current practice

#### Chlamydia-specific double dosage prescriptions as (potential) indicator for PIPT

1.1 The SFK database (‘*Stichting Farmaceutische Kengetallen*’^1^) contains anonymous data on prescriptions covering 95% of public pharmacies (i.e. extramural pharmacies) in the Netherlands. Information is available on the date of issue, delivered amount of tablets, average daily dose recommended to be taken, delivered Defined Daily Dose (DDD); the indication for the prescribed medication is not registered in the SFK. We selected (2004–2015) Azithromycin prescriptions typical and thought to be exclusively used for chlamydia (single dose of 1 g Azithromycin for 1 day or multiple dose thereof), and calculated the proportion of double/multiple dosage prescriptions that could reflect simultaneous PT for chlamydia. This method had been validated before in a previous (unpublished) report, showing that prescriptions of Azithromycin single dose as specific treatment for chlamydia compare well to estimates of the number of chlamydia infections reported nationally.
1.2 The NIVEL-PCD was used to study episode-based data from the electronic patient registration (2015). This network includes over 400 practices, nationally representative by age and gender and by population density and geographical practice distribution.^2^ The database system records data on morbidity, prescriptions and referrals. Analyses were performed with the aim to tabulate single versus double dosages with a diagnosis code for chlamydia infection, as well as repeated prescriptions within a period of 2 weeks after the initial prescription, seen as (potential) cases of PIPT. Chlamydia episodes were identified by ICPC codes (vaginitis (X84), cervicitis (X85) and Pelvic Inflammatory Disease (PID) (X74) in women, and orchitis/epididymitis (Y74) and other genital diseases (Y99) in men) combined with specific chlamydia-prescriptions, based on an earlier developed definition of chlamydia in the absence of a specific ICPC code for chlamydia. [22] Prescription data are ATC-coded. Standard first choice treatment for genital chlamydia is Azithromycin (1 g single dose; ATC-code J01FA10); we also looked at dosages of Doxycycline (2 dd 100 mg 7 days; J01AA02), given for anal infections or epididymitis/orchitis and Amoxicillin (during pregnancies, 3dd 500 mg 7 days; J01CA04). We studied the dosage prescribed, based on frequency (times per day), unit, total number of units prescribed and whether it was a repeat prescription. Episodes with one of the ICPC codes and one of the ATC codes were selected. Episodes with no prescription of antibiotics were assumed not to be chlamydia; those with missing information on dosage were excluded from the analyses.

#### Consultations

1.3 Sentinel practices (*N* = 42) of NIVEL Primary Care Database (NIVEL-PCD), collect data specific for STI-consultations. GPs are requested to routinely complete a questionnaire for each registered new disease episode in a patients’ electronic record with an ICPC-code (International Classification of Primary Care (ICPC-1)) concerning STI/HIV issues. This contains, with anonymous identification numbers, information on testing and diagnosis, patient demographics, reason for consultation and sexual risk behaviour. Since January 2015, questions concerning PN and PT were added; we used 2015 data for the current analyses.

#### Questionnaire data on current practice of PN/PT and attitude towards PIPT

1.4 For a large pre-conference survey, GPs were asked to fill in an online questionnaire at the time of registration for a national GP conference (November 2015). This survey included a question on partner notification of current and ex-partners for patients diagnosed with STIs.
1.5 At the same national GP conference, a short questionnaire was handed out on site to GP participants. This contained questions on the most common methods for PN and PT in the general practice.
1.6 In the NIVEL-PCD, a vignette study on practice and attitude of GPs towards partner management was performed. An annual online questionnaire is distributed among GPs in this network. We included in this questionnaire (in 2016) two case descriptions or ‘vignettes’, known as a useful way to measure reported and/or intended physician practices in an outpatient setting. [23, 24] The vignettes described two hypothetical chlamydia positive patients: (i) a ‘low risk’ case, woman with a steady relationship (25 years, Dutch; partner patient in the same practice), chlamydia probably acquired through an incidental casual partner and (ii) a presumed ‘higher risk’ case: young man (17 years, migrant background), who had 4 different female partners in the last 4 months, and was still seeing one of these. We asked for the GPs intention for PN and PT and his/her attitude towards PIPT.

## Results

### Prescription data

**Fig. 1:**
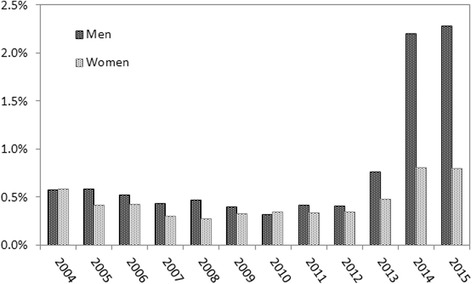
Proportion of double dosages of Azithromycin (1 g single dose) prescribed in public pharmacies in The Netherlands 2004–2015, in men and women

1.1 In total, 443,512 chlamydia-specific prescriptions of Azithromycin (1 g 1d) were registered in the SFK between 2004 and 2015 (increasing, average 37,000 per year). Of these, 99.3% had a single dosage prescription and 0.7% a double or (incidental) triple or more, which may have been direct partner treatment. A clear increase in double dose prescriptions was observed from 2013 onwards: it rose from below 0.5% to 1.5% in the period 2013–2015, much more obvious in men than in women (see Fig. 1).

1.2 For the pilot study on potential PT in the NIVEL PCD, data from 311 general practices in 2015 was available. We identified 18,599 episodes with one of the five relevant ICPC codes; for a large part of these no antibiotic was prescribed which were therefore assumed not to be chlamydia infections and excluded. For 902 chlamydia episodes (in 891 patients), a total of 1078 chlamydia-related antibiotic prescriptions were recorded with dosage information. The major part of the prescriptions was Azithromycin, a smaller part Doxycycline and a few Amoxicillin (with specified dosages or a multiple dose thereof, see Table 1). Double dosages were prescribed in 2.1% of Azithromycin-treated chlamydia episodes: in 2.8% of women and 1.3% of men (12/427 women and 5/372 men; difference not significant; 3 cases gender unknown), most commonly in the age group 30–39 years (3.1%; 6/191) versus 2.1% in <20 years (2/97) and 2.1% in 20–29 years old (9/420).
  Furthermore, double dosages were found for 31.1% of Doxycycline episodes and in none of the Amoxicillin-treated ones. The double-dosed Doxycycline were prescriptions of 28 dosages instead of 14 dosages, unlikely to be PT, probably prescriptions for 2 weeks instead of 1 week, as indicated for epididymitis caused by chlamydia (but duration was not consistently reported). Double-dosages were indeed more common among men than among women (46.2% of men treated with doxycycline and 11.1% of women).
  The proportion of chlamydia episodes with repeated prescriptions was 12.6%, of which 7.6% was registered on the same day (66/902). Of the total, 11.9% was treated with the same antibiotic and 0.8% with a different antibiotic. These proportions did not relate to age group or gender.

**Fig. 2:**
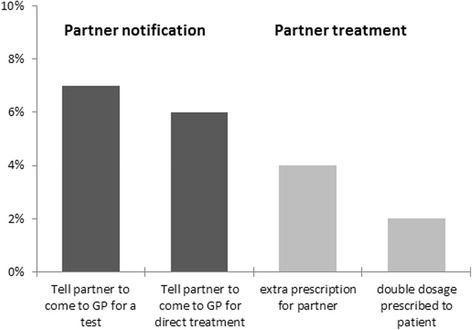
Proportion of 94 chlamydia-positive cases seen by GP for whom one of the specified partner treatments was applied (Sentinel Practices, NIVEL PCD 2015)

1.3 In the 42 sentinel practices, 100 chlamydia cases were recorded in 470 STI-consultations (reported in 2015). The most common way for GPs to inform the patient of his/her chlamydia infection was by phone (*n* = 65) or personal consultation (*n* = 29). The remaining cases heard it from another care provider (under whose responsibility they were tested; *n* = 5) or no follow-up was done (*n* = 1), as the GP stated ‘the patient had already been treated blind, so no follow-up visit was needed’. PN was discussed with the 94 cases contacted: most GPs (*n* = 85) left it to the patient to take further steps; for two cases PN was done by the GP or assistant, while two cases were referred to PN services (five no answer). For 72 of the 94 cases (77%) GPs indicated they did not treat the partner, while 12 said they advised to tell the partner to come to the practice (13%), either for a test (*n* = 6) or for direct treatment (*n* = 5) or both (*n* = 1); four gave no answer. PIPT was applied in six cases (6.4%): the GPs wrote a direct, extra medical prescription for partners of four cases, while they prescribed a double dosage for two cases to treat partners simultaneously (Fig. 2). No clear correlations were found between patient characteristics (gender, sexual preference, age, reason for consultation) and PN/PT.

**Table 1:** Type of antibiotics used for chlamydia: per prescription and per episode, in 2015

Antibiotics	Chlamydia prescriptions N %		Chlamydia episodes N %		Episodes with double dose N %		Episodes with repeated Ct-prescriptions^a^	
Azithromycin (1 g 1d)	937	86.9	802	88.9	17	2.1	93	11.6
Doxycycline (2dd 100 mg 7d)	123	11.4	106	11.8	33	31.1	20	18.9
Amoxicillin (3dd 500 mg 7d)	18	1.7	15	1.7	0	0	2	13.3
Total	1078		902		50		115	

^a^episodes had multiple prescriptions of the same or different antibiotics (maximum 4 recorded)

### Current practice of PN/PT and attitude towards PIPT

**Fig. 3:**
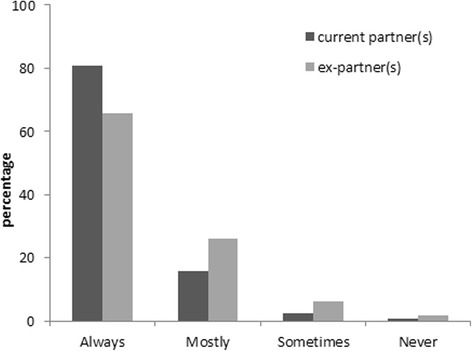
Proportion of 1411 GPs (answering pre-conference questionnaire, Nov 2015) indicating to discuss partner notification with patients diagnosed with an STI, for current partner(s) and for ex-partners

1.4 The online pre-conference survey was filled-in by 1411 GPs (53% of a total of 2650 conference participating GPs). The majority of GPs discusses PN of the current partner and the ex-partner with the patient. Of all GPs, 80% indicated to discuss PN always and 18% mostly with their STI-patients (Fig. 3). The GPs discussed PN significantly less frequently for ex-partners than for current partners (Chi-square *p* < 0.001).

**Fig. 4:**
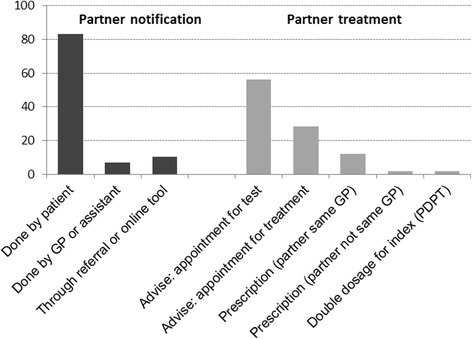
Proportion of 271 GPs (answering ‘multiple choice’ questions at conference, Nov 2015) reporting on most common method used for partner notification and partner treatment for patients diagnosed with chlamydia

1.5 The short hand-out questionnaire was completed by 271 participants at the GP conference. The most common method of PN reported was to discuss this and leave it to the patient to take further steps (83%). In most cases, the GP would advise the patient to tell his/her partner to get tested (56%) or get treated (28%). Direct PT was used for some cases: 4% of GPs treat partners directly: 2% through prescription for the partner and 2% through a double dosage to the index. When the partner is also a patient in the same practice, 12% of GPs write a prescription for him/her at the same time as the index (Fig. 4).
1.6 The vignette study in the annual questionnaire to NIVEL PCD was answered by 289 of 438 persons (response 66%), of which we included the GPs (*n* = 268) for our analysis (21 questionnaires were filled in by GP assistants or managers). The large majority of GPs (87%) indicated they would discuss PN with the patient and leave it to the patient to notify the partner(s) (Table 2). PN via the MHS or another (online) service was more commonly suggested for the high risk than for the low risk case (74% vs 37%). PT advice was via the patient, to inform the partner to consult a doctor to either get tested (76%) or to get direct treatment (55%). PIPT was an option for about one in five GPs: 16% would write a prescription for the partner, while 6% would write a double dosage prescription for the patient to treat the partner simultaneously. In the hypothetical situation that a patient could notify the partner first, prescription for the current partner was considered as an option, more commonly for the low risk than the high risk case (45% vs 24%). Notification and treatment of ex-partners or casual partners was largely left to the responsibility of the patient. In additional comments GPs made clear that PN/PT steps depend on patient- and partner profiles and the character of their relationship (e.g. steady, long-term or casual) as well as the connection between GP and patient.
  Most of the GPs agreed PIPT can be a means to reduce re-infections from chlamydia (62%). GPs said to be open to the idea of PIPT: a considerable proportion of GPs indicated it should be an option for some patients (36%), for most patients (21%) or all (10%). Important advantages were mentioned, especially better infection control and better chances to treat the partner, also practical ones in the sense that it would make PT easier and cheaper. On the other hand, a range of disadvantages were also mentioned. The main objections seem to lie in the uncertainty whether the medication will reach the right person and that there is no chance to give advice to the partner or check his/her risk for allergies or contra-indications. Furthermore over-treatment, potentially leading to resistance, issues on patient privacy, and the fact that the partner may have other STIs or have contact with other partners were mentioned.

**Table 2:** Partner notification and partner treatment practices indicated by 289 GPs for the two ‘vignettes’ of chlamydia cases presented in the questionnaire (NIVEL PCD 2016)

	Case 1: low risk	Case 2: higher risk	Total
Partner notification (PN)^a^			
Discuss with patient, let patient do PN him/herself	93%^b^	81%^b^	87%
Discuss and offer to do PN by GP or assistant	22%	26%	24%
Refer to MHS or use PN website	37%^b^	74%^b^	55%
Partner treatment (PT)a			
Advise to get the partner to consult the GP for a test	72%^b^	81%^b^	76%
Advise to get the partner to consult a GP for direct treatment	52%	59%	55%
Prescribe antibiotics for the partner	18%	14%	16%
Prescribe a double dosage to patient to treat his/her partner	5.6%	6.9%	6.3%
Prescribe AB to partner after patient has done PN	45%^b^	24%^b^	34%

^a^multiple choice questions, more than 1 answer possible

^b^significant difference between low risk and high risk, at *p* < 0.05. PN = partner notification; PT = Partner Treatment

## Discussion

### Findings

This first study about PN and PT of chlamydia in the general practice in the Netherlands shows that GPs currently perform an advisory rather than active, facilitating role in chlamydia PN and PT. In some cases they provide actual PIPT. In general, GPs discuss PN of current and ex-partners with chlamydia patients but leave it to the patient to carry it out. PIPT is applied in an estimated 2–6% of chlamydia cases, based on questionnaires and available electronic consultation- and prescription-data. We found that when the partner is also registered as a patient in the same practice, GPs are more inclined to implement PIPT, i.e. in 12–21% of chlamydia cases. PIPT for unseen/unknown partners is not yet in line with legal regulations.

Follow-up of partner notification and treatment in general practice needs more attention. GPs appeared to be open to the idea of PIPT, although they also expressed concerns, disadvantages and risks. PIPT was an option for about one in five GPs. PN/PT intentions may depend on the STI-risk behaviour of the patient: a high-risk case (with multiple recent partners) would be referred to (online) PN services more often than a low risk case, while a low-risk case with a steady relationship and a partner registered in the same practice was more likely to receive PIPT, especially if the patient would have notified the partner first. PN/PT steps also depend on the connection between GP and patient.

### Strengths and limitations

The strength of our study was the use of multiple data-sources (questionnaires, consultation- and pharmacy data) and the access to representative groups of GPs for questionnaire surveys (via the large national GP conference and the NIVEL-PCD network). We have combined data on intentions and opinions with estimates of PN/PT from GP registers and thereby obtained much better insight into practice and attitude towards PN and PIPT.

Some sources provided more in-depth information than others. The questionnaires used for the (pre)conference surveys were short and with the instruction to choose one ‘most applicable’ answer (multiple choice), more subtle nuances may have been lost. The vignette study on the other hand offered more room for opinions and thoughts, giving insight in opportunities and challenges foreseen for PIPT implementation. Intentions to use PIPT seemed higher than actual practice, maybe due to the discrepancy between intention and implementation but might also indicate that GPs would like to apply PIPT more often but are restricted by guidelines or regulations. Risk profiles of patients (e.g. sexual preference) are not described in regular patient records, so the actual insight into PN/PT practice of GP’s in high risk groups was limited.

The information on PIPT from prescription data had major limitations. The national pharmacy data showed a small but increasing proportion of Azithromycin prescribed as double dosage, but without the indication for the medication, we cannot be certain these were for chlamydia. Moreover, the recent update of the GP STI guideline (end of 2013), [7] provides a 3rd choice treatment option of Azithromycin 2 g single dose for gonorrhoea, for patients for whom Ceftriaxone is contra-indicated and the resistance pattern unavailable. We saw a clear rise in double dosages from 2013 onwards as well as an unequal gender distribution of the double dosages, which suggests these prescriptions may be for gonorrhoea rather than for chlamydia, because gonorrhoea is seen more commonly in men than in women [1], due to the sexual network (MSM) and symptomatic nature of infections in men, urging them to visit their GP. Probably, the level before 2013 (0.4–0.5%) shows the actual proportion of double dosages for patient and partner prescription. In the NIVEL-PCD, part of the double/repeated prescriptions of antibiotics seems unlikely to be PIPT. Double dosages of Doxycycline were prescribed in more than 30% of cases, especially in men (46%); we suspect these relate to other diagnoses including (acute) epididymitis or PID, for which a prolonged course is indicated. The repeated prescriptions on 1 day may also have been registration artefacts, due to back reporting from pharmacies after patients receive the medication. Hence, to be able to use prescription data to monitor PIPT, more reliable information on the prescription and its indication is required. The registration in the national pharmacy database would be improved by including the indication of prescriptions. Prescription data from the GP electronic patient registration would be improved by adapting software to extract more exact dosage schemes and clarifying whether or not back-reporting from the pharmacy takes place in the one or more of the current registration software systems in use.

### Comparison to other studies and countries

We found that the implementation of PIPT for chlamydia is currently low among GPs in the Netherlands, while GPs showed a higher intention to use PIPT when presented with hypothetical chlamydia cases and after PIPT principles were explained briefly. The low level is not surprising, as in the current legal system in the Netherlands the GP should have contact with the partner, before prescribing antibiotics, to check medical history and allergies and inform about the reason for prescription. [25] In fact, some of the cases of PIPT in the records may have been given without observing the law. In other countries where PIPT is legal, levels of uptake are still relatively low: in the US, in a national survey of more than 3000 physicians who treat patients for STIs, approximately 50% reported ever using PIPT, while only 11%–14% of physicians reported usually or always using PIPT. [26] In New York City, the number of expedited partner therapy prescriptions was found to be low and vary greatly between pharmacies in New York City. [27] In the UK, in a pilot RCT the proportion of partners treated after partner details (medical history and allergies) were communicated via the phone (35%) or at pharmacy (46%) did not exceed that by standard patient referral (45%). [18]

In our vignette study, GPs have shown to be aware of the beneficial effects of PIPT in preventing further transmission and facilitating partner management procedures for chlamydia, as has been assessed in effectiveness studies in other countries (US [13–16], UK [28]) and reviews reporting an overall 20–29% reduction of reinfection. [6, 29, 30] Besides the effect on transmission, PIPT can simplify procedures for the partner, which will make treatment much faster. The few cases that may receive PIPT, according to the present study, appear to be at relatively low risk, with a steady partner who is patient in the same practice. Successful treatment of current partners is most important for preventing reinfection of index cases and reducing further transmission of chlamydia at the population level. On the other hand, also for casual partners, or when it is likely that partners will not seek treatment and are at high risk of infection, PIPT is a good last option or ‘better than nothing’ [31]. Modelling studies by Althaus [32, 33] showed that PN for partners up to 18 months back would identify 10% infections in notified partners.

The GPs in our study also mentioned important barriers to implement PIPT, which have been reported in the literature as well. [30, 31] GPs stated in the questionnaire that PIPT reduces the opportunity for the partner to see a professional and get further STI counselling and advice. [34] Current legislation requires direct contact with partners. It remains unclear if contact by telephone is sufficient to meet the legal requirements for the prescription of a drug. It is defendable that under certain conditions contact by telephone with a partner will suffice, but the applicable law nor the existing case law gives a decisive answer to that question. Contacting partners via internet would however certainly require a change in legal requirements. Current GP guidelines on partner notification should be extended with a specification of how and when PIPT can be applied (for partners in the same practice and/or when timely treatment is impossible without PIPT). A further concern is that the patient may not reach his/her partner(s) so partner uptake is not guaranteed. [19, 35] The partner may have other STIs or have sexual contact with other partners, which would not be traced with PIPT. [36] Potential over-treatment by PIPT is a real concern, as only 35% of those notified for chlamydia are found infected. [1] Following antibiotic stewardship, careful use of antibiotics is advocated and embedded in the Dutch habit of relatively low use of antibiotics in patient care in general (the lowest in Europe [37]). Resistance of chlamydia to Azithromycin is not an urgent threat, [38] but for gonorrhoea, a common co-infection with chlamydia which could go unnoticed with PIPT, it is on the rise. [39] Furthermore, in case of an anal (co)infection, chlamydia should be treated with Doxycycline and not just Azithromycin. [7, 8]

## Conclusions/recommendations

GPs in the Netherlands are open to implement PIPT for some chlamydia cases but not for all, provided there are clear guidelines, including clarity about legal options, and indications how PIPT can be applied and which patients and partners would be eligible for PIPT. We advise recommendations for partner treatment in GP guidelines to be extended with a specification of when and how PIPT can be applied.

## Abbreviations

APT: Accelerated Partner Therapy
ATC: Anatomical Therapeutic Chemical classification system
EPT: Expedited Partner Therapy
GP: General Practitioner
ICPC: International Coding Primary Care
MHS: Municipal Health Services
PIPT: Patient Initiated Partner Therapy
PN: Partner Notification
PT: Partner Treatment
STI: Sexually Transmitted Infection

## Footnotes

1 See: https://www.sfk.nl/english (accessed may 2017)

2 See: www.nivel.nl/NZR/zorgregistraties-eerstelijn (accessed may 2017)
